# Combined Effect of Activated Carbon Particles and Non-Adsorptive Spherical Beads as Fluidized Media on Fouling, Organic Removal and Microbial Communities in Anaerobic Membrane Bioreactor

**DOI:** 10.3390/membranes11050365

**Published:** 2021-05-18

**Authors:** Daeeun Kwon, Theo Y.C. Lam, Minseok Kim, Giin-Yu Amy Tan, Po-Heng Lee, Jeonghwan Kim

**Affiliations:** 1 Department of Environmental Engineering, Program in Environmental and Polymer Engineering, Inha University, Inharo 100, Michuholgu, Incheon 22212, Korea; kwonde15@gmail.com (D.K.); asmm1207@naver.com (M.K.); 2 Department of Civil Engineering, The University of Hong Kong, Pokfulam, Hong Kong; yctheolam@gmail.com (T.Y.C.L.); gyatan@hku.hk (G.-Y.A.T.); 3 Department of Civil and Environmental Engineering, Imperial College London, London SW7 2AZ, UK; po-heng.lee@imperial.ac.uk

**Keywords:** anaerobic fluidized bed bioreactor, GAC, ABS media, energy reduction

## Abstract

The combined effect of acrylonitrile butadiene styrene (ABS) spherical beads and granular activated carbon (GAC) particles as fluidized media on the performance of anaerobic fluidized bed membrane bioreactor (AFMBR) was investigated. GAC particles and ABS beads were fluidized together in a single AFMBR to investigate membrane fouling and organic removal efficiency as well as energy consumption. The density difference between these two similarly sized media caused the stratified bed layer where ABS beads are fluidized above the GAC along the membrane. Membrane relaxation was effective to reduce the fouling and trans-membrane pressure (TMP) below 0.25 bar could be achieved at 6 h of hydraulic retention time (HRT). More than 90% of soluble chemical oxygen demand (SCOD) was removed after 80 d operation. Biogas consisting of 65% of methane was produced by AFMBR, suggesting that combined use of GAC and ABS beads did not have any adverse effect on methane production during the operational period. Scanning Electron Microscope (SEM) examinations showed the adherence of microbes to both media. However, 16S rRNA results revealed that fewer microbes attached to ABS beads than GAC. There were also compositional differences between the ABS and GAC microbial communities. The abundance of the syntrophs and exoelectrogens population on ABS beads was relatively low compared to that of GAC. Our result implied that syntrophic synergy and possible occurrence of direct interspecies electron transfer (DIET) might be facilitated in AFMBR by GAC, while traditional methanogenic pathways were dominant in ABS beads. The electrical energy required was 0.02 kWh/m^3^, and it was only about 13% of that produced by AFMBR.

## 1. Introduction

Media fluidization is one of the key aspects to determine the performance of AFMBR in the treatment of low strength wastewater such as domestic sewage [1,2,3]. The media materials fluidized by bulk recirculation alone through AFMBR provides a surface area for cell growth [4,5,6,7,8,9,10,11,12,13]. While AFMBR is widely applied in wastewater treatment along with the production of renewable energy in the form of methane, membrane fouling results in the loss of performance of a membrane due to the deposition of rejected wastewater constituents. GAC particles in AFMBR are often used to provide mechanical cleaning along the membrane surface to reduce membrane fouling [6,14].

While GAC particles can be an excellent tool as fluidizing media [15,16,17], the particles can be broken very easily by frequent collisions due to their weak rigidity during long-term reactor operation. This phenomenon should be more pronounced with larger media size, a higher recirculation rate or mixing intensity through the AFMBR reactor [18]. As a result, GAC particles that are ground into a smaller size can accelerate the membrane fouling because the particles should be deposited on membrane surface or even within membrane pores due to the formation of very dense cake layers easily [18,19,20,21]. Fluidizing the media accounts for the major portion of the energy in the operation of AFMBR, but it can depend strongly upon their intrinsic properties [5,22,23]. For example, higher specific gravity of the media such as GAC than the bulk wastewater present in the reactor, which is often higher than 2.0, requires more energy needed for the media fluidization than smaller ones. Given that the packing ratio of GAC particles is lower, recirculation flowrate through the reactor will be reduced, and thus less GAC particles are expected to be abrased during media fluidization. Nevertheless, membrane fouling is still severe if the upward flowrate is not enough for the GAC particles to cover the whole surface area of the membrane.

Significant efforts were made to apply various scouring agents such as zeolite [8,12,13] and plastic agent [24,25,26] alternatives to the GAC in AFMBR, but only the usage of single media has been considered. Moreover, the use of inorganic particles is still of concern for practical applications because the particles are most likely abrasive in suspension to the membrane. The ABS is a cost-effective, polystyrene-based polymer composite material that has been used widely for various industrial applications [27,28,29]. The advantage of ABS can hold excellent mechanical stability and chemical resistance. The ABS-based materials should employ a low deformation rate. As a result, they can maintain intrinsic properties suitable for long-term reactor operation, even under harsh environmental conditions [30]. Additionally, the ABS beads have lower specific gravity than the GAC, thus requiring low energy consumption for their fluidization. However, the smooth surface of ABS beads provides less surface area for the growth of biofilm than that by GAC [31,32,33]. The AFMBR was operated using plastic beads as single fluidized media for domestic wastewater treatment application [24]. It was reported that bulk volatile suspended solid (VSS) concentration was about 4–5 times higher than those reported with the AFMBR system using the GAC, where most active microorganisms can be grown.

The objective of this study was to combine the GAC particles and ABS beads having the different specific gravity and surface properties as fluidized media while having similar sizes to investigate whether their combined usage is complementary and their microbial communities on each media are similar in the AFMBR treating low-strength wastewater. Specifically, this study examined whether combining ABS beads with GAC could have a synergistic impact on the AFMBR performances such as fouling control, organic removal efficiency and methane production. Although the characteristics of GAC as a biocarrier has been widely researched [34], little is known about the capacity of ABS media to act as a biocarrier and fouling mitigation in the presence of GAC with the AFMBR system. Previous studies have primarily focused on the elimination of biofilm on ABS instead of biofilm development, as ABS is commonly used in medical devices [35,36,37]. Thus, we considered that there is a need to study whether ABS can support methane production from a microbial perspective. In addition, the use of plastic beads was applied to reduce the membrane fouling in the membrane bioreactor, but only single media has been used as a scouring agent to clean the membrane [38,39]. Key questions, such as whether ABS beads could harbor the appropriate microbial community for methane production and whether biocarrier materials would also influence such community formation, hold important implications for reactor performance. Therefore, it is necessary to investigate the impact of using combined usage of media on microbial compositions and energy requirements, as well as reactor performance, which is the key to successful AFMBR operation under the dual media fluidization.

## 2. Materials and Methods

### 2.1. AFMBR Operation

**Figure 1:**
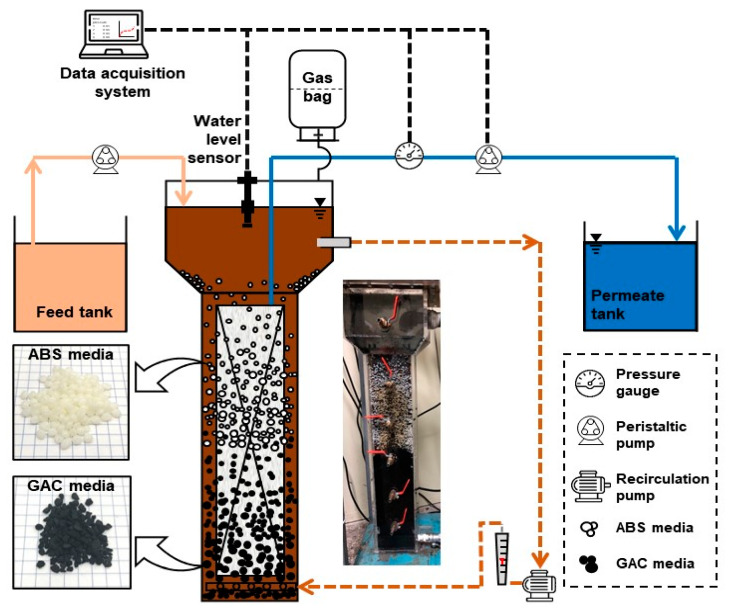
Schematic diagram of AFMBR with the dual media process.

A laboratory-scaled AFMBR system was developed as shown in Figure 1 and applied in this study. The reactor was made using acrylic materials with a total reactor volume of 4 L. A flat-tubular ceramic membrane consisting of alumina dioxide with a pore size of 0.5 um and an effective area of 0.1 m^2^ was applied. The membrane was submerged into the AFMBR and operated by a peristaltic pump (Green Tech, GT-150d, Suwon-si, Korea) at constant permeate flux. A sedimentation tank was installed at the upper part of the membrane reactor to prevent the fluidized media from entering into the recirculation pump (PAN WORLD, NH-150S, Ibaraki-ken, Japan). A recirculation pump was installed at the bottom of the reactor for recirculating bulk suspension from the top of the settling tank to the bottom of the membrane reactor to allow both media to be fluidized along the membrane surface at 3 L/min (0.028 m/s). Synthetic feed wastewater was prepared by using sodium acetate and sodium propionate with 300 mg/L of chemical oxygen demand (COD). The 30 mg/L of ammonium nitrogen and 1 mg/L of phosphate was prepared by using ammonium chloride and potassium phosphate, respectively. Sodium bicarbonate with 100 mg/L was injected into a feed solution to maintain a neutral pH. A level sensor was installed to maintain the water level in the reactor by controlling the feed pump. The reactor was seeded by adding 200 mL of biomass, which is taken from the anaerobic digester operated at the local sewage treatment plant. A Supernatant was added with a 100 mL volume of 1% *v*/*v* into a feed tank to provide trace nutrients for the growth of microorganisms. A Tedlar bag was installed at the top of the reactor to collect biogas produced by AFMBR operated at room temperature.

### 2.2. Selection of Fluidized Media

In this study, two types of media of GAC particles (Calgon Carbon, FILTRASORB 300, Altoona, PA, USA) and ABS plastic beads (Terluran, GP-35, Frankfurt am Main, Germany) were applied as fluidized media in AFMBR. Table 1 shows the characteristics of each media used in this study. The specific gravity of GAC particles and ABS plastic spherical beads was about 2.0 and 1.04, respectively. The size of ABS beads and GAC was about 2.5 and 1.5 mm on average, respectively. Each media was added at 25% of packing ratio into the AFMBR reactor. Since the ABS plastic beads have a lower specific gravity, ABS could be fluidized above the GAC particles at a constant bulk recirculation rate. In other words, the bottom and the top half of the membrane area was covered by GAC particles and ABS spherical beads fluidized, respectively, as demonstrated in Figure 1.

### 2.3. Operation of AFMBR

Table 2 summarizes the operational conditions of the AFMBR during 180 d reactor operation. Operational periods were classified into three phases, where different HRTs were applied. The recirculation flow rate was fixed at 3 L/min to avoid the overflow of ABS plastic beads from the reactor. In Phase I, the HRT was maintained at 8 h during the initial three months of operation, which corresponded to 5.3 L/(m^2^h) of set-point flux. In Phase 2, the HRT was reduced to 6 h by increasing the permeate flux to 7.1 L/(m^2^h). After 20 d reactor operation in Phase 2, the operational mode of the membrane was changed by activating 1 min membrane relaxation every 9 min membrane filtration for 70 d (Phase 3). Membrane relaxation was performed by maintaining the media fluidization along the surface of the membrane without producing treated effluent (permeate).

### 2.4. Microorganism Analysis

Samples of bulk suspended liquid, GAC and ABS media were collected on day 168 during the steady-state condition, which is near the end of the operation. Samples were preserved with ethanol and stored at −20 °C prior to DNA extraction. DNA extraction was performed using PowerSoil DNA Isolation Kit (Qiagen, Venlo, Netherlands). 16S rRNA gene sequencing was performed using Illumina HiSeq with universal primer sets 515F (5′-GTGCCAGCMGCCGCGGTAA-3′) & 909R (5′-CCCCGYCAATTCMTTTRAGT-3′) for bacterial amplification and 519F (5′-CAGCMGCCGCGGTAA-3′) and 806R (5′-GGACTACVSGGGTATCTAAT-3′) for archaeal amplification. Paired-end raw sequences were processed using QIIME 2 v2020.6 [40]. High-quality sequences with a minimum quality score of 30 were obtained using DADA2 pipeline [41] for denoising. Taxonomic classification was performed using pre-fitted sklearn-based taxonomy classifier [42] with a SILVA 138 Ref NR database [43]. Visualization of the microbial composition was done using Phyloseq [44]. Shannon index was selected to evaluate the alpha diversity. Principal Coordinate Analysis (PCoA) was selected to evaluate the beta diversity using unweighted UniFrac distance metrics.

### 2.5. Analytical Methods

In this study, SCOD and volatile suspended solids (VSS) of the bulk suspension, and membrane permeate were measured by Standard Method [45]. The SCOD concentration was measured after filtering the sample through a 0.45 µm pore size using cellulose nitrate membrane filter (Whatman, CAT no.7184-004, Maidstone, UK). The VSS concentration was measured by using a 1.2 µm glass filter (Whatman, GF/C, CAT no.1822-047, Maidstone, UK) according to the Standard Method (No.2540). The removal efficiency (Rc) was calculated according to the following equation.

(1) $$R_{C}=(1-\frac{\mathrm{CP}}{\mathrm{CF}})\times 100\%$$

where C_F_ is the concentration of the feed solution and C_p_ is the concentration of the permeate solution, respectively. Composition of collected biogas was analyzed by using a Hewlett-Packard 6890 gas chromatograph (GC) equipped with a thermal conductivity detector (TCD) and a Hayesep D Packed Column 80/100 (Agilent Technology, Santa Clara, CA, USA); 0.5 mL of gas from Tedlar bag was injected into the GC-TCD by using the 1 mL gas-tight syringe (Hamilton, Reno, NV, USA). The methane production was calculated with a collected biogas volume and methane composition [4]. The surface of each media was observed by using a SEM (SU 8010, Hitachi Ltd., Tokyo, Japan) after platinum coating. The soluble extracellular polymeric substance (EPS) concentration of the bulk suspension was measured by a phenol-sulfuric acid colorimetric method [46]. Here, polysaccharides are assumed to be the major contributor to EPS and hydrolyzed by sulfuric acid to react with phenol. A 50 mL of bulk suspension taken from the reactor was centrifuged at 3200 rpm for 30 min. A deposit of the sample separated with supernatant was then mixed with 2 mL of 5% phenol, after which 10 mL of 98% sulfuric acid was added for about a 10 min reaction period at ambient temperature. Following the reaction, a UV/visible spectrometer measured the absorbance at 490 nm. The absorbance was compared against the calibration curve developed with glucose standard solution prepared in the same way.

## 3. Results and Discussion

### 3.1. Effect of Fluidization of Combined Media on Membrane Fouling

**Figure 2:**
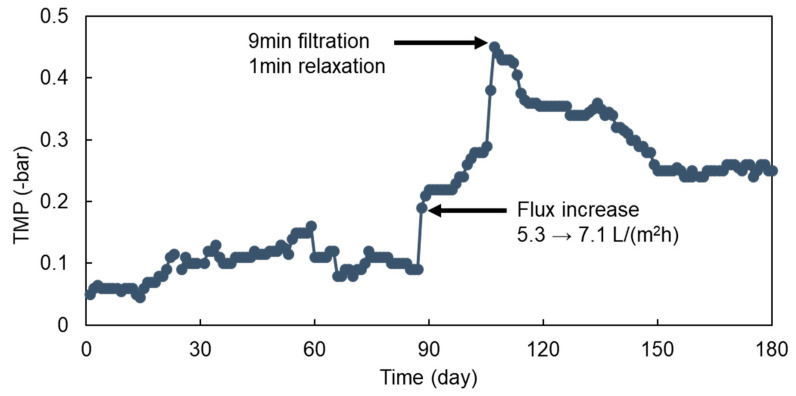
The TMP change of AFMBR with operation time.

Figure 2 shows the change of TMP with the time during the entire operational period of the AFMBR system. During the initial 90 d of operation at 5.3 L/(m^2^h) of permeate flux, which corresponds to 8 h of HRT, the TMP value was maintained at about 0.1 bar. After that, an increase in permeate flux from 5.3 to 7.1 L/(m^2^h) accelerated membrane fouling, showing a rapid increase in TMP to 0.45 bar at 110 d of AFMBR operation. Membrane relaxation was then applied by performing combined media fluidization without producing a membrane permeate for 1 min every 9 min of filtration. After that, the TMP was decreased to the 0.25 bar gradually and then remained during the rest of the operational period. However, further reduction in the TMP value below the 0.25 bar was not observed under the periodic filtration/relaxation at 7.1 L/(m^2^h) of permeate flux.

The reduction in membrane fouling due to the scouring effect caused by the fluidization of GAC or plastic beads in AFMBR has been clearly shown in previous studies [22,24,25,26,38,47,48,49,50,51]. With a flat-tubular ceramic membrane, about 6 L/min of recirculation flow rate was needed in AFMBR for the fluidization of GAC particles at 50 % of the packing ratio. With media fluidization using the same membrane and reactor used previously, due to the lower specific gravity of ABS plastic beads than GAC (1.04 vs. 2.0), the recirculation flow rate needed to be reduced to 3 L/min to avoid the overflow of the ABS beads through the recirculation line. That is, GAC particles could be fluidized along the bottom half of the membrane only, while the above half of the membrane was covered by ABS plastic beads (Figure 1). This fluidized stratification can be expected since the heavier particles require a higher upflow velocity around the particles to cover the whole surface area of the membrane [52]. In other words, the contact with lighter ABS plastic beads on the membrane could result in a less physical impact to reduce membrane fouling.

There is a relationship between the diameter of fluidized media and scouring intensity for the fluidized membrane reactor [53]. With bigger media, a higher recirculation flow rate was needed and this resulted in an improvement of particle motion, thus enhancing the cleaning efficiency on the membrane. In addition, more energy required to fluidize larger media leads to higher critical flux below which membrane fouling does not occur [53]. Reduction in the recirculation flow rate to avoid the overflow of ABS plastic beads through the reactor decreased a bulk upflow velocity along the membrane, and thus the fouling mitigation efficiency could be decreased.

### 3.2. AFMBR Treatment Efficiency

**Figure 3:**
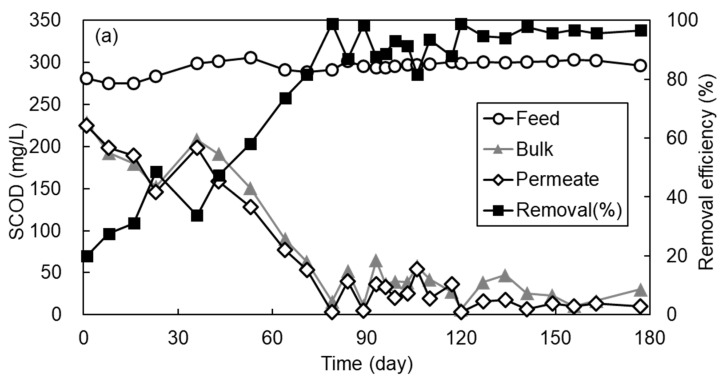

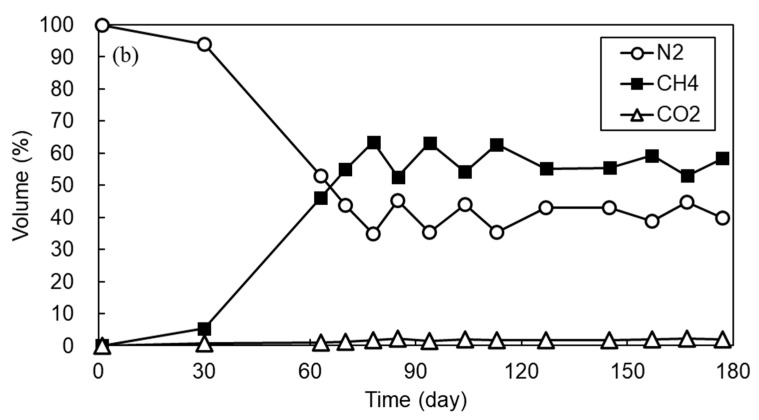
The variation of (**a**) SCOD and (**b**) biogas proportion for 180 days of AFMBR operation.

Figure 3 shows the variation of SCOD concentration and biogas proportion in permeate and its removal efficiency with time observed during 180 d of AFMBR operation. During the initial 30 d of operation, the SCOD removal efficiency was only 20 to 40% due to the period required for microbial acclimation and the small rejection efficiency of organic components by porous MF membrane as applied in this study. After 40 d of operation, the SCOD removal efficiency started to increase gradually and then was maintained at more than 90%. During the initial 30 days of operation, no biogas was produced from the AFMBR, as shown in Figure 3b. At day 60, methane composed approximately 45% of the biogas and then increased to about 65% after day 80. In addition, more than 90% of SCOD removal efficiency was achieved and stabilized at this operational period, suggesting that combined media fluidization consisting of GAC particles and ABS plastic beads did not provide any adverse impacts on the organic removal efficiency. The SCOD in bulk suspension and membrane permeate were almost similar, suggesting that most of the dissolved organic compounds should be removed by biodegradation rather than membrane filtration.

Table 3 shows the mean value of AFMBR performance for each operational period. As a set-point flux was 5.1 L/(m^2^h), the bulk VSS concentration was maintained as 370.8 mg/L on average under which the TMP value was only about 0.1 bar, probably due to the scouring action of combined media to the clean membrane. Furthermore, it was also found that a higher permeate flux resulted in a lower bulk VSS concentration. As the flux increased to 7.1 L/(m^2^h), the bulk VSS concentration reduced to 60 mg/L, but the TMP jumped to 0.45 bar. A possible explanation for this is the transport of VSS present in reactor bulk toward the membrane surface could be more pronounced at a higher permeate flux, resulting in a higher fouling rate [21,54]. In other words, fluidizing plastic ABS beads and GAC particles together along the membrane surface was not very effective to reduce the fouling rate at 7.1 L/(m^2^h) of permeate flux as applied in this study.

After performing the membrane relaxation, bulk VSS concentration was increased significantly to 211.7 mg/L, but TMP was reduced to 0.25 bar. This can support the fact that the media fluidization with GAC and ABS beads on membrane without conducting the permeation is effective to detach the foulant materials from the membrane. The VSS concentration in membrane permeate was near zero during the whole operational period, suggesting that the VSS should be rejected by the membrane almost completely. The EPS concentration in the bulk suspension measured at the end of the operation was 106.4 mg/L. This value was slightly higher than that measured under the fluidization of single GAC [55], but lower than that measured by plastic bead alone as fluidized media in the AFMBR treating the same synthetic wastewater [24]. Although a direct comparison is difficult, our observation suggests that more biomass can be grown on the GAC particles, probably due to the higher surface area provided, thereby lowering the concentration of EPS in bulk suspension than when only the plastic bead is used. In the first 30 d of operation, no biogas was produced by the reactor. From the 60 d of operation, however, methane composition in the biogas produced by single AFMBR was increased gradually and approached about 63% at 80 d of operation. After reactor stabilization, the methane composition in biogas was maintained at a rate higher than 55% regardless of the change in bulk VSS concentration under combined media fluidization.

### 3.3. Microbial Analysis

**Figure 4:**
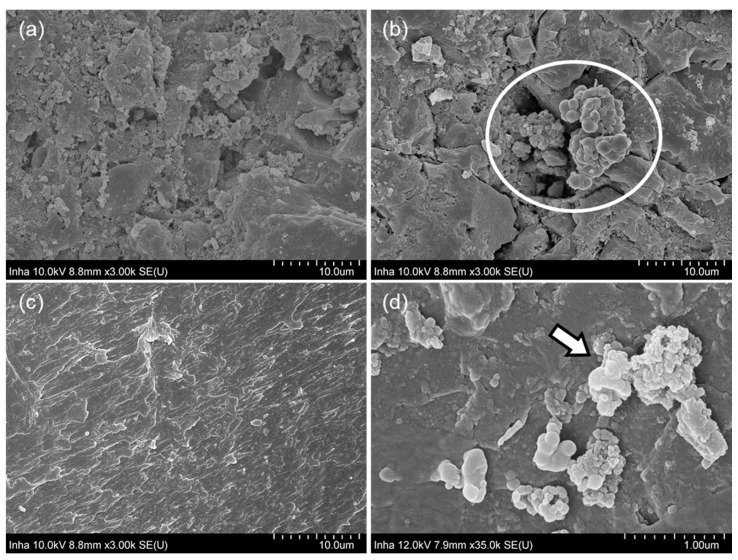
The SEM images of surface on (**a**) GAC, (**b**) used GAC, (**c**) ABS, and (**d**) used ABS media in AFMBR. (**b**,**d**) show some anaerobic microorganisms which adhered and were grown on the surface of each media.

Figure 4 compares SEM images of GAC particles and ABS plastic beads taken from the AFMBR after 180 d of operation. As expected, the surface of bare GAC particles appears to be rougher and more porous than ABS plastic beads (Figure 4a,c). Thus, the biofilm may be grown on the GAC particles favorably, as shown in Figure 4b. Interestingly, there was considerable evidence that the ABS plastic beads provided a surface for the growth of microorganisms with a spherical morphology (Figure 4d). The hydrophobic surface of ABS beads may be involved in the adhesion of microorganisms [56,57,58]. However, more studies are needed to better understand interactive biofilm formation on polymeric materials such as ABS.

To further evaluate the microbial compositions in the combined media, we performed 16S rRNA gene sequencing on bulk liquid, GAC and ABS beads. The samples were collected on day 168, during which the biogas production and methane composition had reached a steady state. Thus, the samples can represent matured microbial communities. Significant differences in the number of clean sequences between GAC (archaeal: 4788; bacterial: 49918) and ABS beads (archaeal: 1681; bacterial: 15033) were observed, while bulk (archaeal: 4817; bacterial: 57211) and GAC were comparable. Considering that the samples were processed using the same protocol, it is most likely that the low sequence count is attributed to the low DNA recovery from the ABS media. Although the SEM images suggested that microbes adhered to the surface of both media, the 16S rRNA gene data suggested that ABS harbored fewer microbes than GAC.

**Figure 5:**
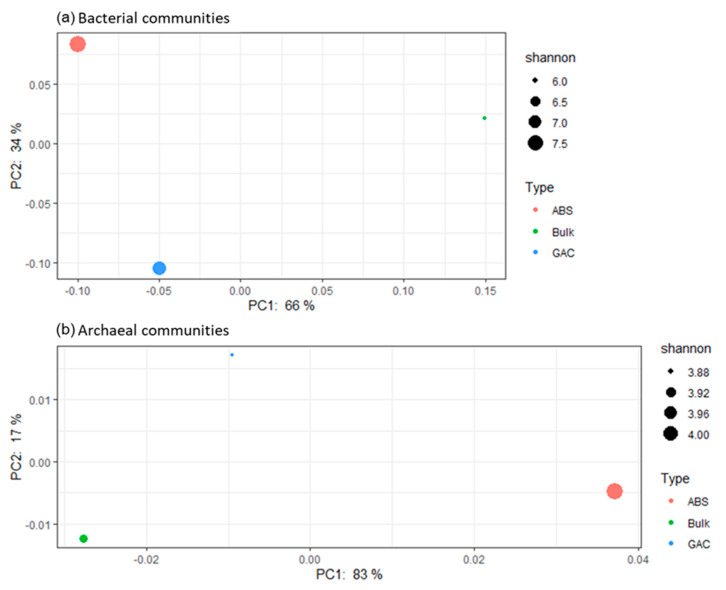
Principal coordinate analysis (PCoA) performed on microbial community structure dissimilarity. A weighted UniFrac distance metric was used to evaluate the compositional difference with the account of the relative abundance distribution. Samples were colored by their sample type. The size of the node was based on the value of the Shannon diversity index, which measures the richness and evenness of the microbial community. Panel (**a**) shows the result of the bacterial communities; panel (**b**) shows the results of the archaeal communities.

The Principal Coordinates Analysis (PCoA) visualized the difference in microbial composition among each sample (Figure 5). The separation among the three samples indicated that their microbial composition was distinctively different. Based on bacterial composition, the fluidized media were more similar than bulk liquid (Figure 5a), and this is mainly attributed to the lower abundance of Proteobacteria on both media (28 to 30%) as compared to bulk liquid (50%) (Table 4). Between GAC and ABS beads, compositional differences (2 to 8%) in the phyla Firmicutes, Patescibacteria, Planctomycetes and Spirochaetes further distinguished the two media. In terms of archaeal composition, on the contrary, GAC and bulk liquid shared a more similar profile (Figure 5b). In particular, the uncultured Ca. Methanofastidiosales was absent in both bulk liquid and GAC, but was present on ABS (4.4%). These observations collectively suggest that biofilm and bulk communities in AFMBR are distinct from each other, corroborating with previous studies [59,60]. Collectively, biocarrier material is also a determining factor in how the microbial community is shaped.

**Figure 6:**
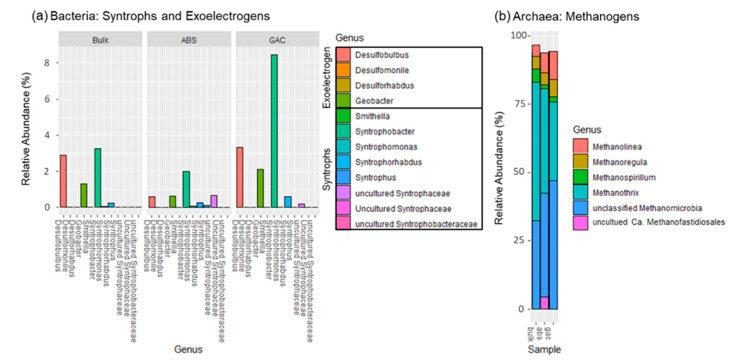
Microbial compositions illustrated in relative abundance at the genus level. Panel (**a**) shows the propionate-degrading syntrophic bacteria and exoelectrogens. Panel (**b**) shows the archaeal communities; non-methanogenic archaea are excluded.

Figure 6 illustrates the distribution of syntrophs, exoelectrogens, and methanogens to focus on how these substrates were consumed in different media. Based on taxonomic classification, the microbial metabolism on GAC and ABS beads were examined and compared. The syntrophs accounted for 9.29% of the bacterial population in the GAC sample, which mostly consisted of propionate-degrading *Syntrophobacter* (8.46%). It also harbored exoelectrogens such as *Desulfobulbus* and *Geobacter* with relative abundances of 3.32% and 2.12%, respectively. The sulfate-reducing *Desulfobulbus* reportedly utilizes propionate for growth and produces acetate under a sulfate-limiting environment, as is the case with our system [61]. This taxon was previously observed in an AFMBR system using polyvinylidene fluoride (PVDF) as a scouring agent and biocarrier [59], but its role in AD metabolism is still unclear. Aceticlastic *Methanothrix* (28.8%) and an unclassified *Methanomicrobia* (38%) were dominant methanogens in the GAC; the remaining methanogenic community consisted of hydrogenotrophic methanogens. Since acetate and propionate were used as carbon sources in the synthetic feed, *Syntrophobacter* was likely to convert propionate into acetate and hydrogen. While the hydrogenotrophic methanogens utilize hydrogen, acetate can then be utilized by *Methanothrix* for acetoclastic methanogenesis. Moreover, *Geobacter* can consume acetate and extracellularly release electrons that are used by *Methanothrix* via direct interspecies electron transfer (DIET) [62]. Under such circumstances, *Methanothrix* could utilize CO_2_ as the carbon source for methanogenesis, which would otherwise be impossible without its exoelectrongenic partner [60]. Although bulk liquid also contains *Geobacter* (1.3%), the currently known mechanisms of DIET requires proximity to function and is unlikely to occur in the suspension of liquid [62].

In the ABS sample, the relative abundance of *Syntrophobacter* (2.01%) and *Geobacter* (0.65%) were lower than in the GAC, suggesting that the syntrophic synergy and occurrence of DIET were relatively limited. While ABS harbored a higher percentage of *Methanothrix* (38.32%), it is most likely to produce methane via the acetoclastic pathway, as the *Geobacter* was in low relative abundance (0.65%). Besides, *Syntrophobacter* only accounted for 2.01% of the bacterial population in ABS beads, and the total syntrophic population is about 3.15%. Such synergy helps to maintain a thermodynamically favorable environment for the degradation of metabolites in the anaerobic digestion system [63]. While ABS beads could act as a biocarrier to facilitate the development of a syntrophic relationship between syntrophs and methanogens, the syntrophic synergy could be limited. It is plausible that compared to GAC, a more extended enrichment period is required for the development of a syntrophic population on ABS beads, although more studies would be needed to verify this.

### 3.4. Energy Requirements

The energy requirement of the pump for operating AFMBR was estimated using a power requirement equation as follows [55]:

(2) $$P=\frac{Q\gamma E}{1000}$$

where P is the power requirement of the pump (kW), Q is the recirculation flow rate of bulk suspension (m^3^/s), γ is 9800 N/m^3^, and E represents hydraulic pressure head loss for fluidization (mH_2_O).

Table 5 shows the calculation of the energy demand and energy production of AFMBR applied with dual media fluidization in this study. Assuming that the efficiency of the pump is 65%, the total energy requirement is calculated as 2.03 × 10^−2^ kWh/m^3^. When converting methane in the generated biogas to electrical energy with an efficiency of 33%, it was calculated as 1.62 × 10^−1^ kWh/m^3^ and the generated amount compared to the consumed amount was 7.97 times higher than the energy required to operate AFMBR. At 6 L/min of recirculation flow rate, as only GAC particles were applied as fluidized media under 50 % of the packing ratio, the total energy required at this condition was 3.09 × 10^−2^ kWh/m^3^ [55]. As mentioned, combined use of GAC and ABS beads requires less energy than that needed by using a single GAC under the same total packing ratio (50%) due to a lower recirculation flow rate required. Therefore, the combined use of both media provides a beneficial effect on reducing the operational costs of AFMBR while the fouling mitigation efficiency may be relatively low.

## 4. Conclusions

Combined media fluidization using GAC particles and ABS plastic beads in single AFMBR exhibited fouling mitigation effectively while limiting the TMP value to less than 0.2 bar at 6 h of HRT. Nevertheless, a high organic removal efficiency (>90%) was achieved with the production of stable methane composition in the biogas as the two solid media were fluidized together. Microbes adhered to both media, but the microbial community was dependent upon the biocarrier material. The number of sequences was similar between GAC and bulk suspension, while fewer sequences were observed for ABS. Additionally, syntrophs and exoelectrogens population were more abundant on the GAC particles than the ABS beads. Therefore, it was more likely that DIET was utilized for methane production in GAC, while the microbes in ABS more heavily relied on traditional methanogenic pathways. The electrical energy required with dual media fluidization was only 0.02 kWh/m^3^, which was 87% lower than the energy produced by AFMBR system.

## Figures and Tables

**Table 1:** Characteristics of fluidized media.

	ABS Plastic Beads	GAC Particles
Specific gravity	1.04	2.00
Diameter (mm)	2~3	1~2 (>0.84)
Surface area (m^2^/g)	0.28	700~1300
Moisture absorption (%)	0.95	2.00
Shape	Flat-sphere	Angular-sphere

**Table 2:** Operational conditions of AFMBR

Period	1	2	3
Day	0~90	91~110	111~180
Flux (L/m^2^h)	5.3	7.1	7.1
HRT (h)	8	6	6
Relaxation			Filtration 9 min Relaxation 1 min
Recirculation rate (L/min)	3	3	3
Temperature (°C)	25	25	25

**Table 3:** The mean value of AFMBR performance during each operational period.

Period	1	2	3
Day	0~90	91~110	111~180
SCOD removal (%)	58.5 ± 28.3	89.3 ± 4.4	95.3 ± 3.2
VSS in bulk (mg/L)	370.8 ± 100.0	60.0 ± 28.9	211.7 ± 92.5
Biogas (L/d)	<1	1	1
Methane (CH_4_, %)	37.1 ± 27.3	60.1 ± 5.1	56.3 ± 2.5

**Table 4:** A heat map of bacterial composition at the phylum level (relative abundance > 1%).

Phyla	Bulk	GAC	ABS
Acidobacteria	0.0081	0.0351	0.0459
Bacteroidetes	0.1964	0.1601	0.0987
Caldiserica	0.0032	0.0187	0.0124
Chloroflexi	0.0851	0.1701	0.1671
Firmicutes	0.0099	0.0162	0.0423
Omnitrophicaeota	0.0053	0.0345	0.0346
Patescibacteria	0.0352	0.1611	0.0768
Planctomycetes	0.0106	0.0197	0.0685
Proteobacteria	0.5090	0.3071	0.2854
Spirochaetes	0.0082	0.0090	0.0364
Synergistetes	0.0031	0.0183	0.0180

**Table 5:** Energy balance for electrical energy requirements and potential production with AFMBR.

Electrical Energy Required	
- Energy for media fluidization and influent AFMBR	
- Reactor head loss (mH_2_O)	1.00 × 10^−2^
- Reactor influent plus recirculation flow rate (m^3^/s)	5.01 × 10^−5^
- Fluidization energy requirement (kW)	4.92 × 10^−6^
- Required pumping energy (kWh/m^3^)	9.84 × 10^−3^
- Energy for permeation (permeate production)	
- Average TMP (mH_2_O)	1.23984
- Permeate flowrate (m^3^/s)	1.47 × 10^−7^
- Permeate energy requirement (kW)	1.79 × 10^−6^
- Required pumping energy (kWh/m^3^)	3.38 × 10^−3^
- Total pumping energy (fluidization + permeation) (kWh/m^3^)	1.32 × 10^−2^
- Total electrical energy required for pumps (fluidization + permeation (kWh/m^3^) (65%)	2.03 × 10^−2^
Electrical Energy Production Potential from Methane	
- Methane production (mol/m^3^ wastewater)	2.23
- Methane energy content (kWh/m^3^) (0.22 kWh/mol CH_4_)	0.49
- Electrical energy production from methane (kWh/m^3^) (33%)	1.62 × 10^−1^
Required/produced energy	12.55%
Electrical energy produced/required	7.97

## Data Availability

Not applicable.
